# Some Remarks on the Surgery of the Upper Eyelid

**Published:** 1886-09

**Authors:** F. Richardson Cross

**Affiliations:** Surgeon to the Bristol Eye Hospital; Ophthalmic Surgeon to the Bristol Royal Infirmary


### SOME REMARKS ON THE SURGERY OF THE UPPER EYELID.

By F. RICHARDSON CROSS, M.B. Lond., F.R.C.S.; Surgeon to the Bristol Eye Hospital; Ophthalmic Surgeon to the Bristol Royal Infirmary.

Each lid fundamentally consists of a fibro-cartilage, attached to the periphery of the orbit by ligaments. The combined ligaments (palpebral, external, and internal tarsal) constitute a continuous membrane, split horizontally by the palpebral aperture, the borders of which are thickened by the free edge of the cartilages, closely covered by conjunctiva. The outer canthus is fixed by the tarsal ligament, whilst the more complicated inner canthus is mainly supported by the tendon of the orbicularis.

The palpebral ligament is in front of the cartilages; it is attached to the superior one only at its lower edge, leaving the upper edge and anterior surface free for the insertion of the levator palpebræ, which thus intervenes between the ligament and the upper part of the cartilage.

Along the anterior free border of the lids the roots of the lashes are attached to the cartilage by a very dense cellular tissue; whilst just in front of the posterior border, which lies against the cornea, are the openings of the Meibomian glands. These glands are closely bound down by conjunctiva upon grooves in the cartilage, and their ducts perforate it to reach the free surface of the edge of the lid.

The arrangement of the mucous membrane upon the inside of the tarsus is quite special; it is unlike that lining the remainder of the lid, and is in marked contrast with that on the eyeball. Its attachment is most intimate and immovable; it is very vascular, red in colour, and showing well-marked papillæ.

The skin of the lids is very thin and delicate; its glands are very small, and the hair-follicles are extremely rudimentary. No proper subcutaneous tissue can be defined between it and the orbicularis muscle, which, together with a few special slips (orbito-palpebral of Sappey), alone intervenes between the skin and the palpebral ligament.

Slight abnormalities of the conjunctiva of the lids cause considerable worry to the patient; and if allowed to become chronic, they may easily affect the epithelium of the cornea or even produce pannus. Removal of the small calcareous "grits," that are not infrequently found, especially perhaps in gouty patients, is easily conducted under cocaine and affords marked relief. Recently I have treated some cases of granular lids with keratitis (trachoma); and have found excellent results follow the continued instillation of jequirity infusion, according to de Wecker's formula (at first twice a day, and then daily), over a period of two or three weeks. In my cases no very severe inflammation was set up; the eyes became tolerant of the drops, and continued to improve under their use. The cure has been usually completed, according to the conditions present, by using some caustic application or by scarifying.

The application of jequirity in a severe case of interstitial keratitis, with ophthalmia, produced very little effect.

Any form of blepharitis deserves immediate attention, for continued inflammation may permanently alter the mucous membrane, together with the accurate shape of the tarsal cartilage, which is so intimately connected to it. Recent cases are rapidly cured by the ordinary methods: painting with a strong solution of nitrate of silver has been the most efficacious remedy in my hands for obstinate cases. If the cure is long postponed, and much ulceration has occurred, the resulting cicatricial tissue may, by its contraction, produce entropion and soon develop severe corneal trouble.

It is not less important to aim at an early cure of eczema affecting the eyelashes; for this, in attacking the roots of the hairs, attacks also the cellular pad on the anterior edge of the tarsal cartilage, from which they grow; so that not only are the hairs degenerated, displaced, or destroyed, but the tarsal cartilage suffers with production of trichiasis, and consequent pannus.

Under such conditions, some operative treatment must be undertaken.

Epilation, unless accompanied by destruction of the displaced hair-follicles, is quite useless as a curative measure; an effort must be made to restore the proper anatomical relation of the parts comprising the lid: if this is impossible, the main consideration must be for the cornea. The incurved cartilage is to be everted by cutting a horizontal groove on its cutaneous surface; and two or three Snellen's knots must be passed vertically between the skin carrying the cilia and the cartilage (just traversing the surface of the latter above and below the groove), through the orbicularis, and out through the ligaments at the brow. If the hairs are much incurved, the horizontal skin incision should be made close above them; the roots must then be freed from the cartilage; and, by threads, the ciliary margin can be advanced forward and upward in front of the cartilage, which may also be grooved in any desirable position. Where, together with trichiasis, the edge of the lid is much altered, it is best to split the latter with a knife, entirely separating the lashes and skin on the one hand from the cartilage on the other. The anterior flap with the hairs may then be drawn upwards to any desirable extent, while the cartilage with the inner edge of the lid and the Meibomian ducts remain *in situ*, or dealt with according to necessity.

Inflammation may so thicken and enlarge the tarsal body as to prevent the proper raising of the upper lid : I have recently seen two well-marked cases of this mechanical ptosis. In a female servant, 25 years old, the upper third of the left cornea was covered by a drooping lid, depending on extra weight and enlargement of a chronic tarsitis. As the thickening gradually subsided under treatment, the ptosis completely recovered. In a girl of 17 a similar condition was predisposed by weakness on both sides of the elevator of the lid : distinct ptosis resulted on the one side, where the muscle was insufficient to raise the thickened cartilage.

Nævus may also cause mechanical ptosis, and obstruct the sight. I have in two cases found electrolysis a good plan of treatment ; if there is much fibrous tissue, the whole is best dissected out. Eversion of the lid, with exposure of the palpebral conjunctiva, may result from unwise interference.

Where lesion of the upper branch of the third nerve is the cause of ptosis, together with paralysis of the superior rectus, care must be taken that improvement of the lid does not cause diplopia.

Over-action of the orbicularis is very rarely the cause of ptosis ; and the operation of removing a horizontal strip of its fibres, with or without skin, is not satisfactory.

It will usually be found that the occipito-frontalis is active in attempting to do the work of the paralysed levator palpebræ.

By engaging the tarsal cartilage in the loop of a Snellen's knot, passing the ends of the thread in front of the palpebral ligament and the edge of the orbit, through the orbicularis and frontalis, and out through the skin of the forehead, the tarsus can be drawn upwards and fastened to the frontalis muscle. This operation, which Pagenstecher recommended at the International Congress in 1881, is thoroughly scientific, and I have found it give good results. The thread used should be somewhat coarse, in order that it may cause some irritation, with development of granulation tissue. I have lately retained it *in situ* for a month, in a case of bilateral congenital ptosis. The inflammatory thickening in subsiding is followed by cicatricial contraction; and a tendinous band is left, which connects the frontalis with the tarsal cartilage.

The tendency to contraction of the tissues of the upper lid is well seen after abscess, and especially if a foreign body has been left undetected, as may easily be the case. I was consulted for cicatricial shortening, with ectropion of the upper lid, by a man, who said that nearly a year before he had stumbled upon an upright stick, the end of which had wounded this lid. Much inflammation followed, gradually subsiding into a discharging sinus. He produced a fragment of wood, nearly half an inch in length, which, to his astonishment, had escaped from the sinus nine months after the accident.

Ernest S., aged five, came to me a week after he had wounded his upper lid by falling on a clothes-peg. There was extreme cellulitis of the lid: this was incised, and treated by cotton-wool pressure. It seemed to heal fairly well, but very slowly; and the opening, which continued to discharge, became adherent to the edge of the orbit. There was great tenderness throughout. Five months after the accident, a rough piece of stick, about half an inch long, was brought me, as having come out of the wound, which had almost closed. The marked tenderness had disappeared; the child was unable to close the eye, which remained open during sleep, by reason of the adhesion of the lid to the orbit. De Wecker reports similar cases. In traumatic inflammation of the lid, most careful search should be made for a foreign body; for if cellulitis reach the orbit, it may result in atrophy of the optic nerve.

